# Many faces of survey equipment failures during marine research at sea—Risk analysis

**DOI:** 10.1371/journal.pone.0272960

**Published:** 2022-08-26

**Authors:** Maria Kubacka, Lucjan Gajewski, Marcin Burchacz, Maciej Matczak, Paweł Janowski, Jakub Piotrowicz

**Affiliations:** 1 Gdynia Maritime University, Maritime Institute, Gdańsk, Pomorskie, Poland; 2 MEWO S.A., Straszyn, Pomorskie, Poland; American University of Sharjah, UNITED ARAB EMIRATES

## Abstract

Research of the marine environment is still a huge challenge for humanity. Each survey campaign is a complex project, where research vessels and relevant survey equipment is used. One of the problems that limit the success of working at sea are failures of survey equipment. The aim of this paper was to identify the most common survey equipment failures during marine research, find their causes and analyze identified risks. The authors employ risk assessment methodology in maritime research at sea and present its practical utility and contribution in social and organizational development. For this purpose we based the analysis on the review of relevant project documentation (Daily Progress Reports, Observation Cards) and the questionnaire addressed to specialists who carry out their survey work on board research vessels and also people involved in the implementation of offshore projects. The research reveals that 76.3% respondents participated in a project which had to be stopped due to a failure of the survey equipment that required return to the port which highlights that the problem which was analyzed is of particular importance. The questionnaire form was designed to obtain as much information as possible on the types of failures with examples and also their causes according to three groups: human factors, technical factors and forces of nature. Twelve risks were identified and analyzed. The authors also stress the relationship between the quality of research project management and its implementation in the context of the failure rate of measuring equipment.

## Introduction

### Context of the study

According to the National Oceanic and Atmospheric Administration [1] nearly 71% of the Earth’s surface is covered with water and oceans which hold about 96.5% of all Earth’s water. More than 80% of our oceans are unmapped, unobserved and unexplored. The investigation of the marine environment still remains one of humanity’s greatest challenges. Maritime industries constitute an important branch of the world economy, which includes: petroleum industry [2, 3], maritime industry [4, 5] and seafood industry [6, 7]. They all require human action at sea supported by research vessels and relevant survey equipment.

Currently the fleet of research vessels (R/V) being crucial assets during the environmental investigation of the marine environment consists of over 880 units worldwide. Despite the fact that vessels are an extremely important element of the survey process, their usefulness is defined by a selection of relevant survey instruments installed onboard. Due to the complexity and diversity of marine environment investigations (e.g. habitat monitoring, geophysical and geotechnical investigations, search for wrecks and artefacts, exploration of mineral resources, assistance in the implementation of investments at sea) as well as variability of operation areas and duration of survey campaigns, proper selection of equipment available onboard is crucial. Achieving an appropriate level of equipment performance efficiency requires its proper selection, both in terms of quantity (redundancy) and quality. In both cases, it is related to the cost of research, therefore the identification of basic failures and their consequences is one of the key elements in the preparation and implementation of offshore projects.

What’s more, equipment performance efficiency depends on the ever-changing sea conditions and weather conditions that influence sensitivity of the equipment and vessels [8] as well as location at a distance from the coast [9]. The success of offshore projects depends on many factors [10, 11], also on the efficient conduct of planned survey campaigns.

The failures themselves may therefore be the result of quality deficiencies in the equipment, but also result from extreme research conditions, and finally from human errors that may occur under such conditions. Therefore, the appropriate assessment of the risk associated with the operated equipment requires identification of basic problems occurring during the research and then estimating their impact on the effective implementation of the project. As a result, it is possible to manage equipment risks and minimise their impact on the costs and timeliness of offshore research.

### Risk of equipment failures in the offshore projects

The concept of risk was introduced to management in 1964 by DB Hertz [12] and gradually accepted in the other areas of the field, including project management [13]. After the identification process, having a complete list of risks, the next step is to proceed to the qualitative risk assessment (QRA). It describes the risks in non-numerical terms and categorises them depending on their importance for the given project and the impact on the level of achievement of the objectives. When prioritizing risks we apply, among others, the probability and impact matrix [14–16]. According to Dziadosz and Rejment [17] or Mahamid [18], it is the most useful method of project risk analysis, identification and initial risk assessment.

A Guide to the Project Management Body of Knowledge [19] defines risk as an uncertain event or condition, that if it occurs, has a positive or negative effect on a project’s objective. Projects in Controlled Environments (PRINCE2) methodology gives the definition of risk consistent with that contained in the Management of Risk (M_o_R) methodology: a risk is an uncertain event or set of uncertain events that, if they occurred, would affect the objectives of the project. BS ISO 31000 defines risk as an effect of uncertainty on objectives that is often expressed in terms of a combination of the consequences of an event and the associated likelihood (probability) of occurrence [20].

According to Olubiyo [21] equipment failure can be any event in which equipment cannot accomplish its intended purpose or task. As an equipment failure risk while working at sea in this paper the authors understand an event or set of events when the survey device does not fulfil its role and the data is not collected or is collected with errors.

When managers do not address risks that have a negative impact on project effectiveness, it may result in various problems such as cost overruns, schedule delays and poor quality of collected data. Thus, relevant assessment and management of risk becomes a significant element of the project development and implementation. The more complicated and sensitive the project is, the more attention should be paid to risk management methods and tools. The investigation of the marine environment is regarded as a particularly demanding area of economic activity, so relevant standards have been developed.

An international standard for the safe management and operation of ships and for pollution prevention is specified by the International Management Code for the Safe Operation of Ships and for Pollution Prevention (ISM Code) [22]. A safety management system (SMS) that fulfils the objectives of the code should be established and implemented by shipowner or charter. One of the main requirements of the code is a risk assessment. According to IMO [22] all identified risks to vessels, personnel and environment should be assessed and appropriate safeguards should be established by the company. In order to ensure the highest level of safety for the implementation of marine research, risk assessments of functions on board and the tasks performed are conducted. Before starting any research activity, each staff member is obliged to read the document describing the associated risks. It must also be noted that the process of risk assessment and management should not only be correctly formulated as well as implemented but should also be regularly evaluated so that the objectives of the code are achieved. However, despite following the recommended procedures, accidents and failures do happen.

When analyzing the above it becomes clear that addressing risks should be based on analysis of information gathered from relevant people directly involved in implementation of surveys at sea. The authors goal was to obtain the greatest amount of information about the causes of failures and what factors affect the failures. Understanding the root cause of an event is key to preventing reoccurrence and addressing any existing issues with operating procedures, equipment design, maintenance practices and other.

Completely separate from ISM Code is the assessment of risks associated with the management of the survey process, including the management of risks associated with the use of specialised survey equipment during offshore survey work. Due to the very specific and niche nature of this type of activity, there is no uniform standard. Therefore, it would be useful to create dedicated systems for assessing and managing risk in the marine survey process.

### State of knowledge

The issue of equipment failures and related risk assessment is widely discussed in literature [23–26], however no specific researchers devoted to identification of the marine research equipment failure and associated risk have been identified. Narrow specialisation of such activities with a dedicated subsea equipment structure, the scientific basis of research, commonly disregarding their economic efficiency, as well as marginal importance of the research phase (being the pre-investment part of project implementation) in the maritime investments (from the investor perspective), are only the basic factors of this situation.

In literature the marine equipment failure is mostly related to the offshore oil & gas industry, where economic or environmental consequences are elaborated and predicted by selected methods or models [27, 28]. Furthermore, the technical aspects of failures of offshore processing equipment and quantitative approach to risk (QRA) are the main considerations [29]. Researchers are also traditionally focused on the technical aspects of failure and risk [30]. In some research, however, additional factors are investigated. The concepts of human reliability analysis (HRA) approaches incorporate human performance and the resulting human errors in QRA for a more holistic overview of the associated risks with offshore facilities [31]. Thus, the STAMP-HFACS methodology can express interactions between people, technical equipment, and the environment [32]. The complex quantitative risk modelling methodologies can also commonly reflect and analyse specific factors with respect to human, operational and organisational risk influencing factors [30].

Investigating the causes and effects of failures in offshore measurement equipment, as well as the risks associated with these events is, therefore, an area that requires attention. This is especially important for organisations and enterprises carrying out sea floor research, both for science and industry.

### Research objectives

This study focused on the following research objectives: (a) identification of the most common survey equipment failures while working at sea, (b) finding causes of survey equipment failures, (c) identified risks assessment, (d) creating response plan for the identified survey-equipment failures risks.

A scarce peer-reviewed research was found in a literature review regarding the more specific risks associated with survey equipment failures while working at sea. This paper therefore attempts to fill this research gap by presenting an identified risk and its analysis that can be used in the risk identification process when planning implementation of the offshore projects. The authors decided to use unique data set of specific project documentation and results from the questionnaire conducted among surveyors working on the research vessels and other people directly involved in the implementation of offshore projects. Such data hasn’t been previously used, which we confirmed after literature review.

This study comprises of the following parts:

- We discuss the general problems and risks associated with survey equipment failures during marine research at sea;
- We review documentation which allows identifying the most common survey equipment failures which took place during project implementation and divide them according to the cause of the problem: human factors, technical factors and forces of nature (independent of human influence);
- To get more detailed information, there was prepared a questionnaire for people working on research vessels and involved in the implementation of projects at sea;
- All the identified weather-related risks were assessed, quantified and qualified;
- The final part of the paper discusses identified risks and their causes as well as limitations of the research and recommendation for future investigations.

## Materials and methods

### Risk identification

According to Pritchard [14] risk identification is an organized and detailed activity aimed at detecting specific types of risk occurring in a given project, which is also a key stage in the whole risk management process. In this paper risk related to failures of survey equipment was identified. The necessary information was collected based on the information-gathering techniques (questionnaire) and the documentation review of the offshore investment projects implemented in cooperation with the Maritime Institute—Gdynia Maritime University and MEWO S.A. Both companies are located in Gdańsk, Poland.

#### Documentation review

For the purpose of this paper risk related to failures of survey equipment was identified. One of the techniques used was a documentation review which consisted of careful analysis of the relevant survey vessel’s documents, with a clear goal to identify the risks that may arise during the project implementation [14]. We analysed two types of documents: daily progress reports (DPR) and observation cards. The DPRs are filled in by the party chief and sent to the project manager every day for the ongoing control of work implementation and progress. The reports cover 24 hours and include, among others, the current position of the vessel, short descriptions of work performed on board, conducted operational works at sea, and a summary of work planned for the following 48 hours. In the DPR form a party chief comments and informs about events such as accidents, incidents or near misses. Information which were of particular interest included the number and duration of research vessels downtime caused by survey equipment breakdown as well as information about the type of failure.

The second type of documents that were analysed were observation cards which are an important element of continuous analysis and observation of current activities that take place in projects. This document was created as part of Integrated Management System and ISO 9001, 140001, 45001 (Lloyds Register) policy to allow observation and assessment of behaviour and activities of the vessel survey team. Observation cards are available to everyone involved in the implementation of the project, including the ship’s crew and survey team. Using these documents all irregularities can be reported with details such as: location of occurrence, information if the event was related to their own personnel or contractors, give full event description and its causes as well as propose a solution.

**Fig 1:**
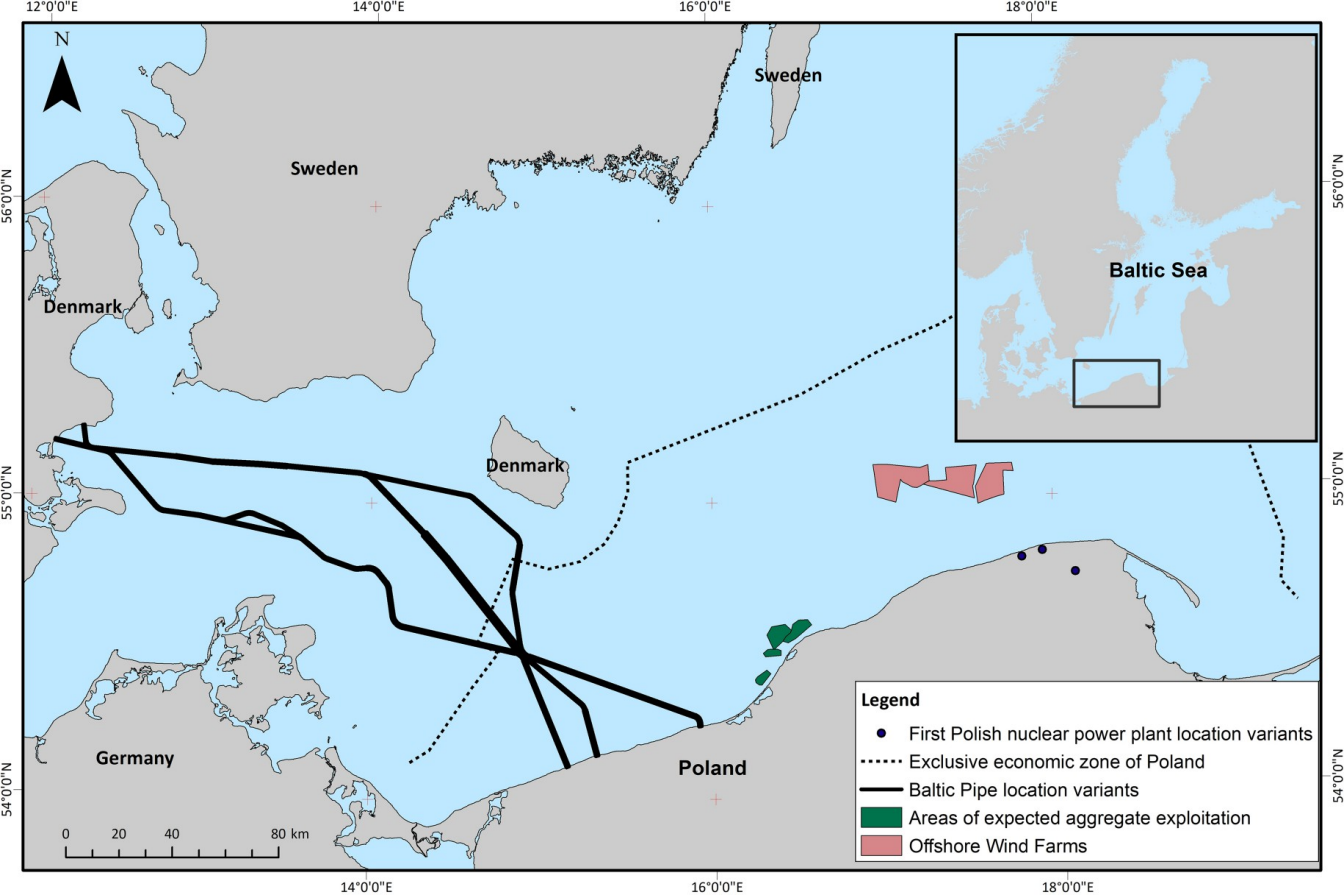
Map with marked areas for offshore investments where Maritime Institute and MEWO conducted pre-investment research used in the documentation review for this paper.

In order to obtain information for the purposes of this article, 36 observation cards from the selected projects implemented by the Maritime Institute in 2019–2021 and the DPR from the seabed research projects carried out by the Maritime Institute and the consortium of Maritime Institute and MEWO S.A. were reviewed [33–38]. The research areas are indicated on the map below (Fig 1). The analysis of failures from the observation cards allowed to determine the frequency of occurrence of a given failure and its consequences for the project and delays for the schedule (duration of the failure).

#### Three factors contributing to equipment failure

On the basis of the above-mentioned documents, it was possible to identify the most frequently reported failure causes, which we divided into three groups:

1. human factor,
2. technical factor and
3. forces of nature.

According to Başar et al. [39] human factors cover all of the actions revealing the relation between people and machines. Another definition says that human factors refer to environmental, organisational and job factors, as well as human and individual characteristics which influence behaviour at work in a way which can affect health and safety [40]. In the literature, we also find terms such as human error [41] and human element which, according to IMO, is recognized as a key element of the safety of life on board ships and a contributing factor to most of the casualties in the shipping sector. We took all of these into account as a human factor. Technical factors cover all technical issues which make survey conducting impossible. The forces of nature while working at sea are mainly related to the weather and waves which limit and make work at sea difficult. The factors also include all elements of the environment that affect the survey equipment and quality of measurements.

#### Questionnaire

Documentation review was the basis for creating a questionnaire that was to provide as detailed information as possible on the most common causes of survey equipment failures and specific examples of survey set breakdowns. In addition, the same set of events related to equipment failures was analyzed in terms of dependence of their occurrence on the possibility of their prevention through proper project management. For the paper’s purposes, the completely voluntary online survey on failures of measuring equipment was created and distributed among stakeholders as the bilingual Google-form (English version plus Polish translation). The survey was not approved by any IRB/ethics committee as it was anonymous and based on the answers given, participants (including their contact details/emails) could not be identified. The respondents have not been approached live, therefore no physical contact, risk of discomfort, inconvenience or psychological distress could have occurred.

The online survey consisted of a combination of both open and closed questions including rankings and choices of multiple answers.

The structure of questionnaire included:

- The initial information about questionnaire respondents’ qualifications (surveyor, crew member, other—to define) and years of experience (four interval scales to choose from: less than one year, 1–5 years, 5–10 years and 10+ years)
- Question on participation in the project which had to be stopped due to a failure of the research vessel (Yes and No—i.e. positive and negative answer)
- Question on participation in project works which had to be stopped due to a failure of the survey equipment and vessel was required to return to the port (Yes and No—i.e. positive and negative answer)
- Both positive and negative answers lead to the next section on the main issues related to the failures of measuring equipment.

In the first two open questionnaire respondents had to describe: 1. the most common survey equipment failures encountered while working at sea; 2. the most memorable witnessed equipment failure onboard a vessel and cause of this failure.

The next part of the survey was dedicated to ranking the most common cause of measuring equipment (human error, forces of nature, technical factor) with scale from 1—the least common to 5—the most common).

The last section of the survey consisted of a few obligatory questions which used several survey methods: open questions, rankings of importance and questions of choices.

In terms of the most common human factor contributing to equipment failures, respondents could mark multiple answers (lack of caution, lack of qualifications or good training, rush, no compliance with the procedures, fatigue, poor work organisation) and add their own answer in the “other” section. The same method was used for the most common technical factor contributing to equipment failures (choice between two answers and “other” text to fill-in).

The survey ended with two open questions: list/description of witnessed failures of survey equipment caused by the forces of nature and question on sea basins where respondents operated.

The request to fill in the online survey was addressed primarily to surveyors, crew members, and other persons involved in performing research at sea. It was distributed internationally via email in March and April 2021 between involved stakeholders: surveyors, scientists, analytics and crew members e.g. IMOR research vessel crew and analytics, surveyors from Maritime Institute and the other research bodies, private companies such as MEWO S.A, International Research Ship Operators (IRSO), Polish Register of Shipping (PRS), and European Research Vessels Operators (ERVO) consisting of members from countries such as Belgium, Denmark, Finland, Germany, France, Italy and Spain. Some parties were asked to forward the link to the questionnaire in order to receive the largest number of results possible. In total, 200 individuals were approached of which 76 answered the survey which makes it a proper representative sample. The first answer was given on 31 March 2021 while the last one was received on 21 April 2021.

### Risk assessment

**Fig 2:**
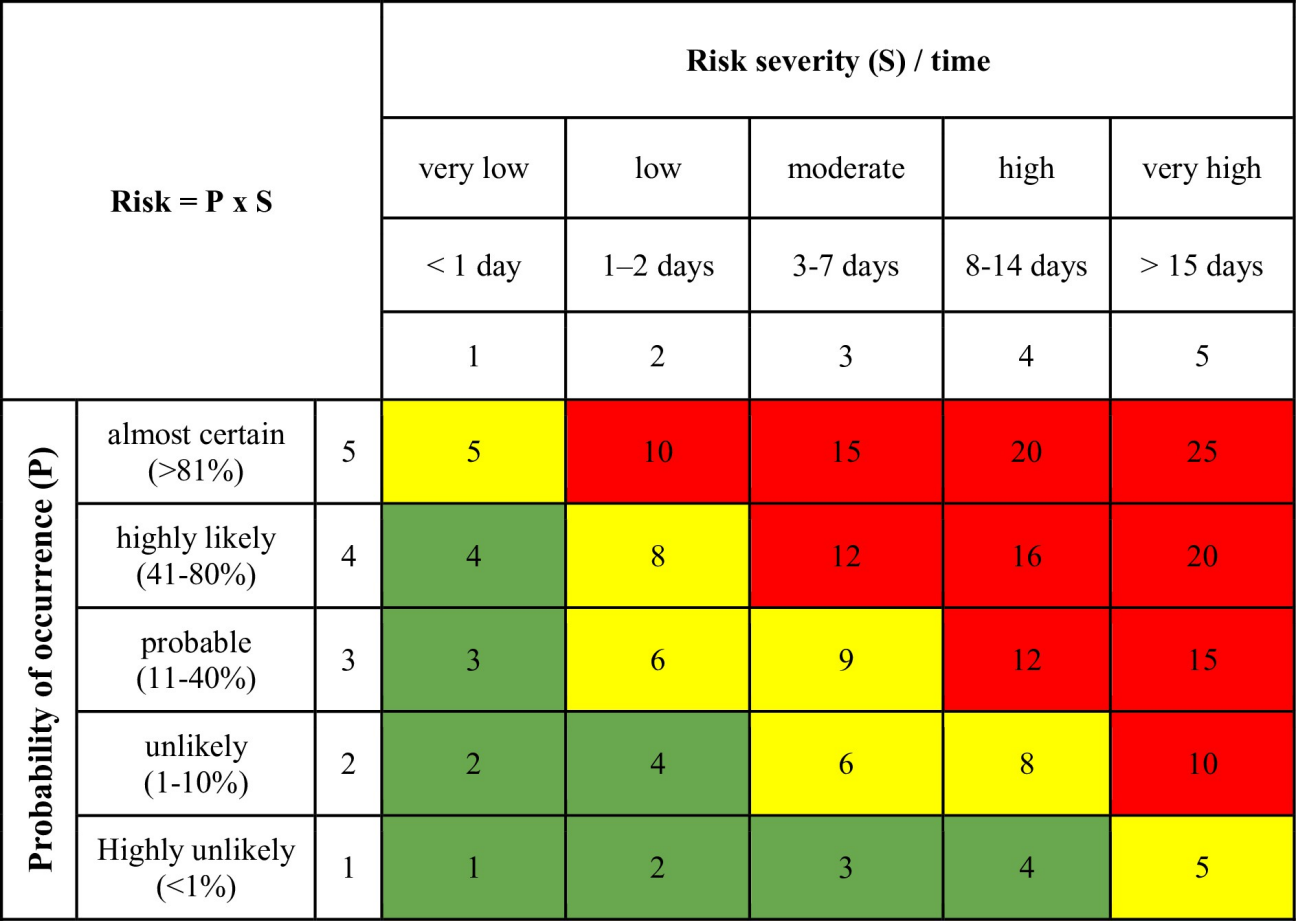
Risk classification matrix (source: Internal data).

In order to assess the risk associated with measuring equipment failures, a probability and consequence class assessment risk matrix was developed for the delays in completion of survey works at sea in relation to the work schedule. Probability of risk occurrence and impact on the work schedule were determined in a five-scale dimension for each type of identified risk on the basis of the analysed documents, answers provided in the survey and our subjective assessments [42]. The probability of the risk occurrence was determined in percentage. Highly unlikely events (less than 1% chance) are not expected to occur, but cannot be excluded. Unlikely events (11–30% chance) mean such an event occurred in the past and cannot be completely ignored. Probable events (31–60% chance) occurred in the past (but are not common) and even though the conditions for the implementation of the present project are different, they are quite a real possibility. Highly likely events (61–90% chance) occurred several times in the last few years, while almost certain events (more than 81% chance) occurred frequently in previous projects. The hazard severity was determined in terms of its consequences on the project schedule and time delays. Consequences of very low impact were defined as delays of less than 1 day. Low impact was determined as 1–2 days delay, moderate impact as 3–7 days delays, high impact as less than 2 weeks delay and very high impact as more than 2 weeks. Risk (R) is calculated as a combination of potential hazard Severity (S) and Probability (P) of occurrence of this hazard according to the following formula R = P × S (Fig 2).

The evaluation of the degree of impact and probability of occurrence of identified risks was based on information obtained from documentation review, questionnaires and our experience in offshore projects. The basis for determining the risk severity were DPR documents, according to which it was possible to estimate the duration of each failure and the time needed to repair inoperative equipment or find other solutions to continue the survey. Risk probability was assessed after the study of listed documents and questionnaire responses (the question about the most common survey equipment failures encountered while working at sea). Assessment of the risk probability was made based on the frequency of information on a given failure in the documents and in the interviewees’ responses. For example, in the DPRs, information about cable malfunction appeared very often, therefore risk probability was defined as highly likely. Based on the same set of documents, we found that the repair of such a failure never lasted longer than 2 days, therefore the risk severity was defined as low. Due to confidential clauses in the contracts we are not allowed to publish detailed data from the analysed observation cards and DPRs.

Green zone (1–4) is a low risk which is acceptable. Yellow zone (5–9) is a significant risk that can hardly be accepted. Risk at this level is tolerated only if further risk reduction is not possible. The red cells (10–25) indicate a high risk that is unacceptable. Such a risk calls for counteractive measures. A risk classified in the highest group (red cells) is of top priority and the information on it must be passed to the upper—level management officers, e.g. the project council, the contract manager on the ordering party’s side, or even the investor. Work should not be started until the risk is minimised. If it is not possible to reduce the risk, work can not continue. Before initiating a project at sea and before mobilizing the equipment, the contracting party should provide the contractor with project documentation containing risk assessment analysis so that the contractor is aware of the risk involved from the very beginning and is able to introduce appropriate preventative measures.

## Results

The questionnaire was completed by 76 people, most of whom were employed as surveyors (52.6%). The second largest group of respondents were technicians (15.8%). Crew members and scientists formed groups of seven people (9.2%). The questionnaire was also filled in by: analysts and sample takers (5.3%), survey managers (2.6%), r/v managers (2.6%) and one post-processor (1.3%). Respondents were well experienced in offshore works as 64.5% of them had been working at sea for longer than 10 years, a group of 15.8% have worked at sea for 5 to 10 years, while 19.7% for the period of 1 to 5 years. None of the respondents had worked at sea for less than a year. The Baltic Sea was indicated by the respondents as the main location for research. However, only 15 people indicated the Baltic as the only location of their research.

### Causes of survey equipment failures

Among all the respondents 76.3% participated in a project which had to be stopped due to a failure of the survey equipment which required return to the port.

**Fig 3:**
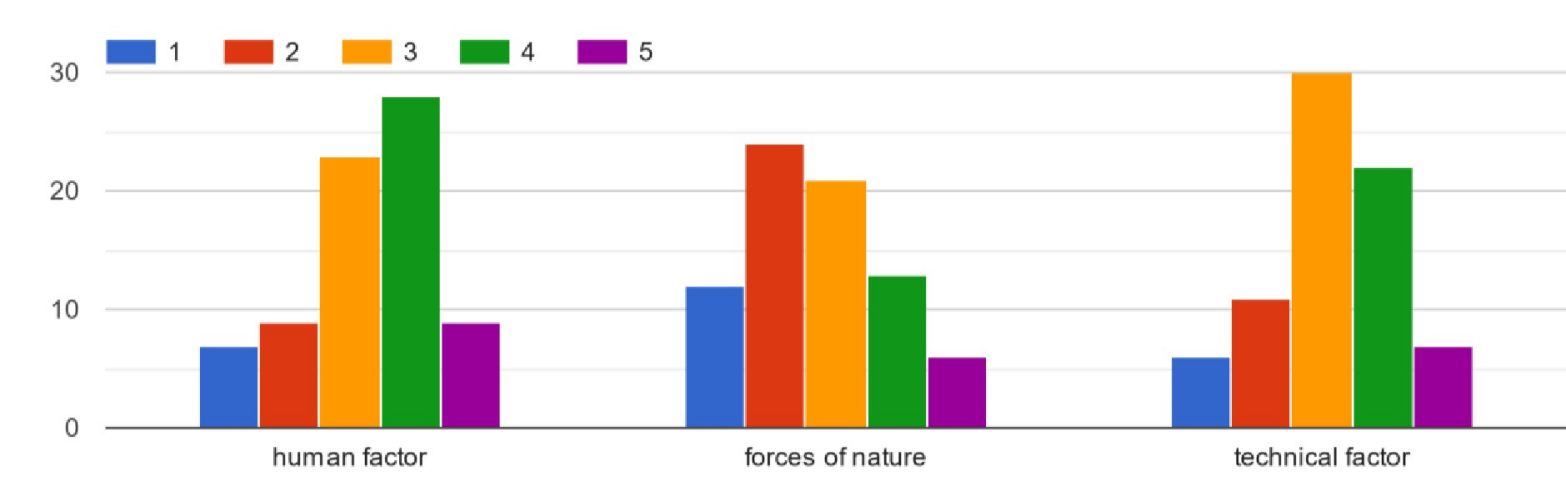
The most common causes of measuring equipment failures assessed from 1—the least common to 5—the most common.

The most common cause of survey equipment failures was assessed as a human factor (Fig 3), 9 respondents pointed it as the most common and 28 as frequent. The technical factor was assessed as the second most important cause of equipment failures, 7 respondents indicated it as the most common, 22 as frequent. The most often given answer (30) was moderately common. Rare (24) and the least common (12) cause of survey equipment failures according to respondents are forces of nature.

#### Human factor contributing to equipment failure

**Fig 4:**
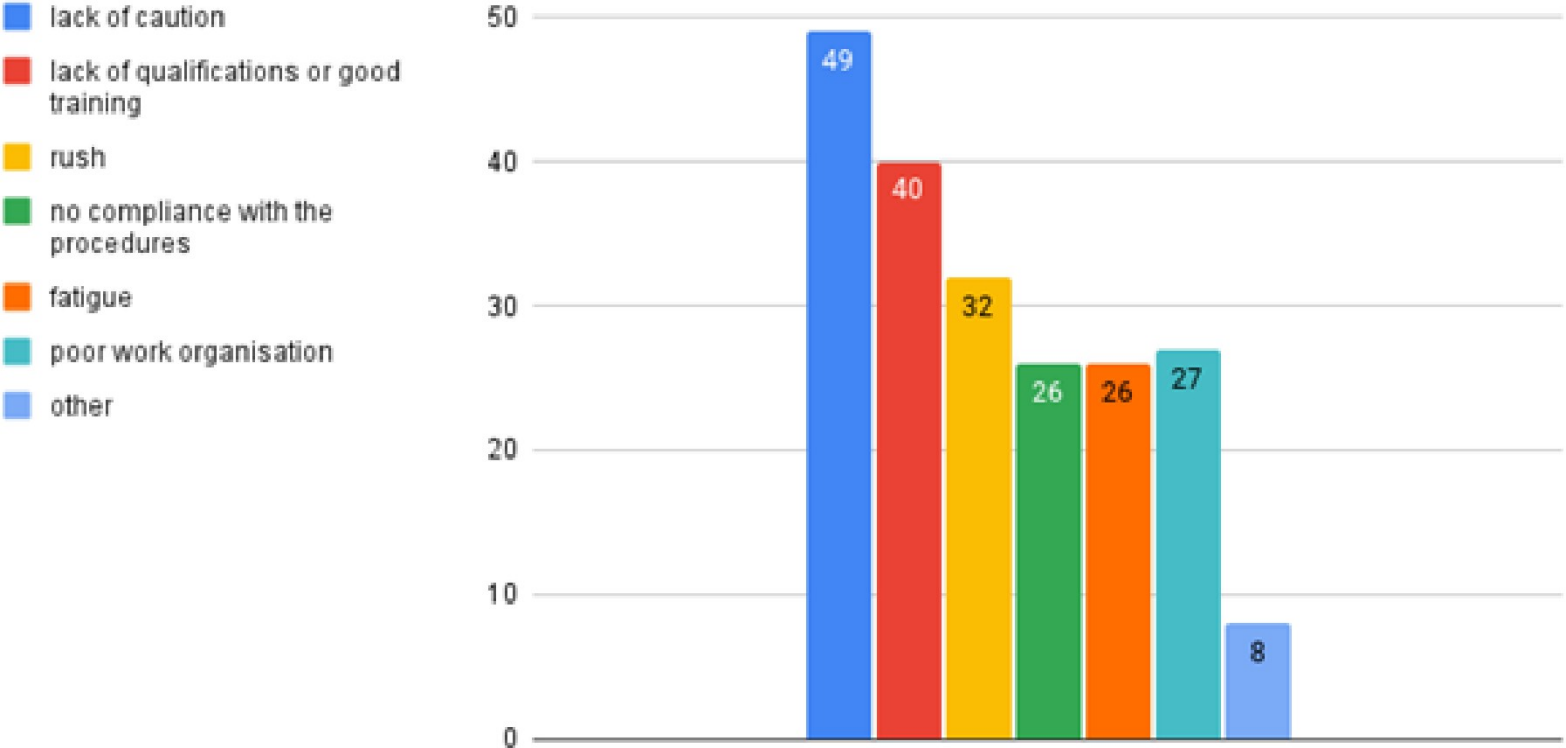
The most common causes of measuring equipment failures due to human factor according to interviewees.

The respondents indicated that the most common cause of failures due to human error was lack of caution, which was pointed out by 49 interviewees. The second most common cause was lack of qualifications or good training (40 indications), the third was rush (32 indications). Poor work organisation was pointed out by 27 respondents, no compliance with the procedures and fatigue was selected by 26 respondents. Other causes of failures related to the human factor were given by eight respondents (Fig 4).

Examples of other causes of failures due to human factor named by the respondents are listed below:

- working under stress;
- lack of communication between the survey team and the vessel crew;
- risky vessel manoeuvres;
- lack of experienced personnel on board;
- circumstances beyond control;
- a false belief that using different survey method than the one recommended by the experienced supervisor will bring better results;
- poor supervision;
- poor design of new survey instruments;
- poor understanding of the marine environment in which the equipment is deployed.
- cumulation of many factors;

#### Technical conditions contributing to equipment failures

**Fig 5:**
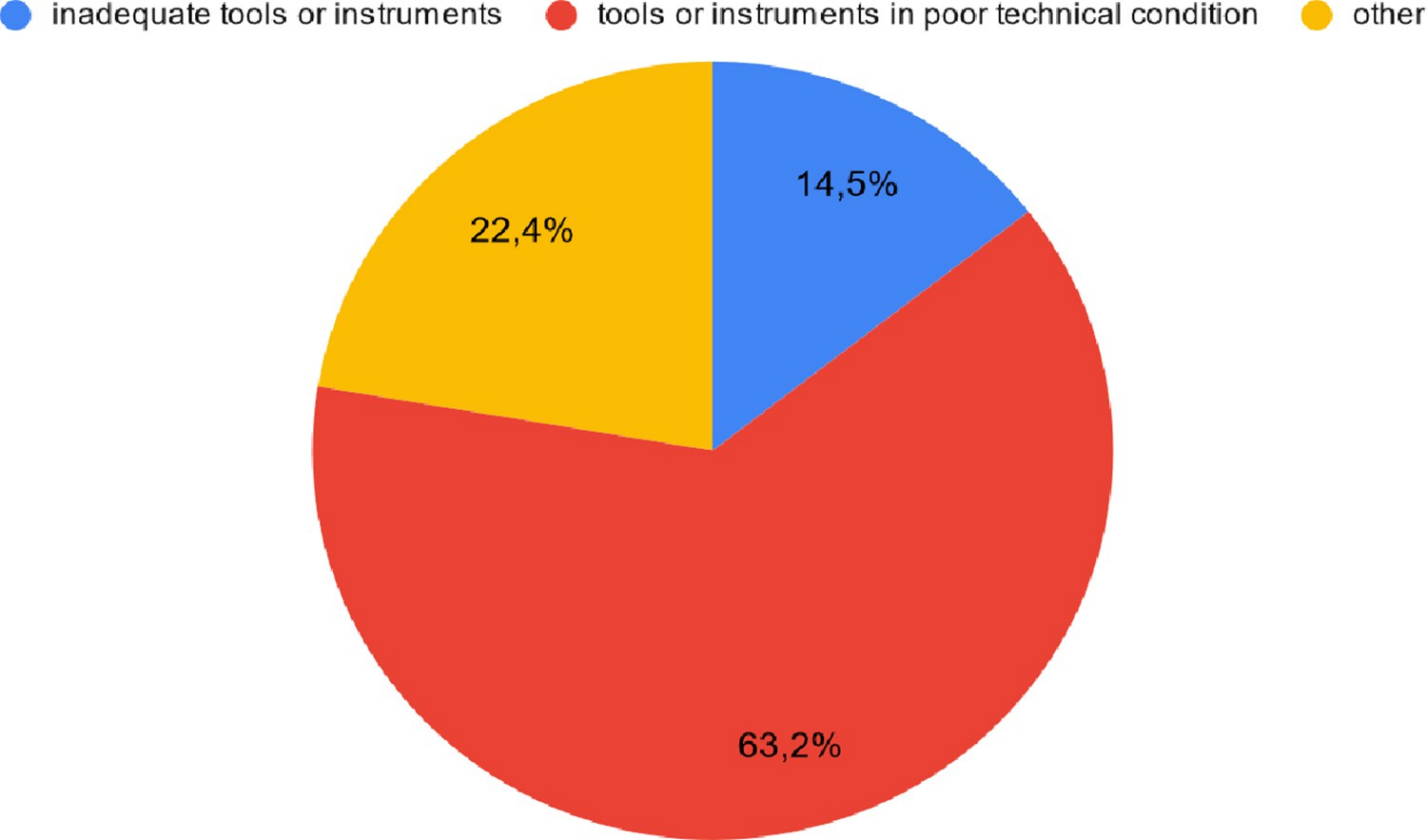
The most common causes of measuring equipment failures due to technical conditions according to interviewees.

Poor technical conditions of tools or instruments were indicated by 63.2% of respondents as the most common technical factor contributing to equipment failures. Inadequate tools or instruments were pointed out by 14.5% of interviewees. Other causes of failures related to the technical factor were mentioned by 22.4% of respondents (Fig 5).

Examples of other causes of failures related to technical conditions named by the respondents are listed below:

- intense usage;
- space dedicated to equipment not meeting the requirements of ABP;
- lack of regular inspections and instrument testing before taking the measurements;
- wear of materials;
- inappropriate vessel for research;
- equipment failure in the course of normal usage despite proper installation and repair e.g., disconnection / tear of cable connecting the measuring; equipment, dislocation of equipment part, etc.;
- deterioration of individual elements from usual usage;
- circumstances beyond control;
- wear and tear;
- pushing limits of equipment capabilities;
- material stress, extension of equipment life period over factory;
- recommendations;
- poor maintenance, inadequate maintenance;
- lack of redundancy;
- inexperience with newly designed equipment or systems ("Bleeding Edge");
- poor IT infrastructure;
- contamination in the work environment interfering with the survey results;
- poorly selected or installed equipment.

#### Forces of nature contributing to equipment failures

**Fig 6:**
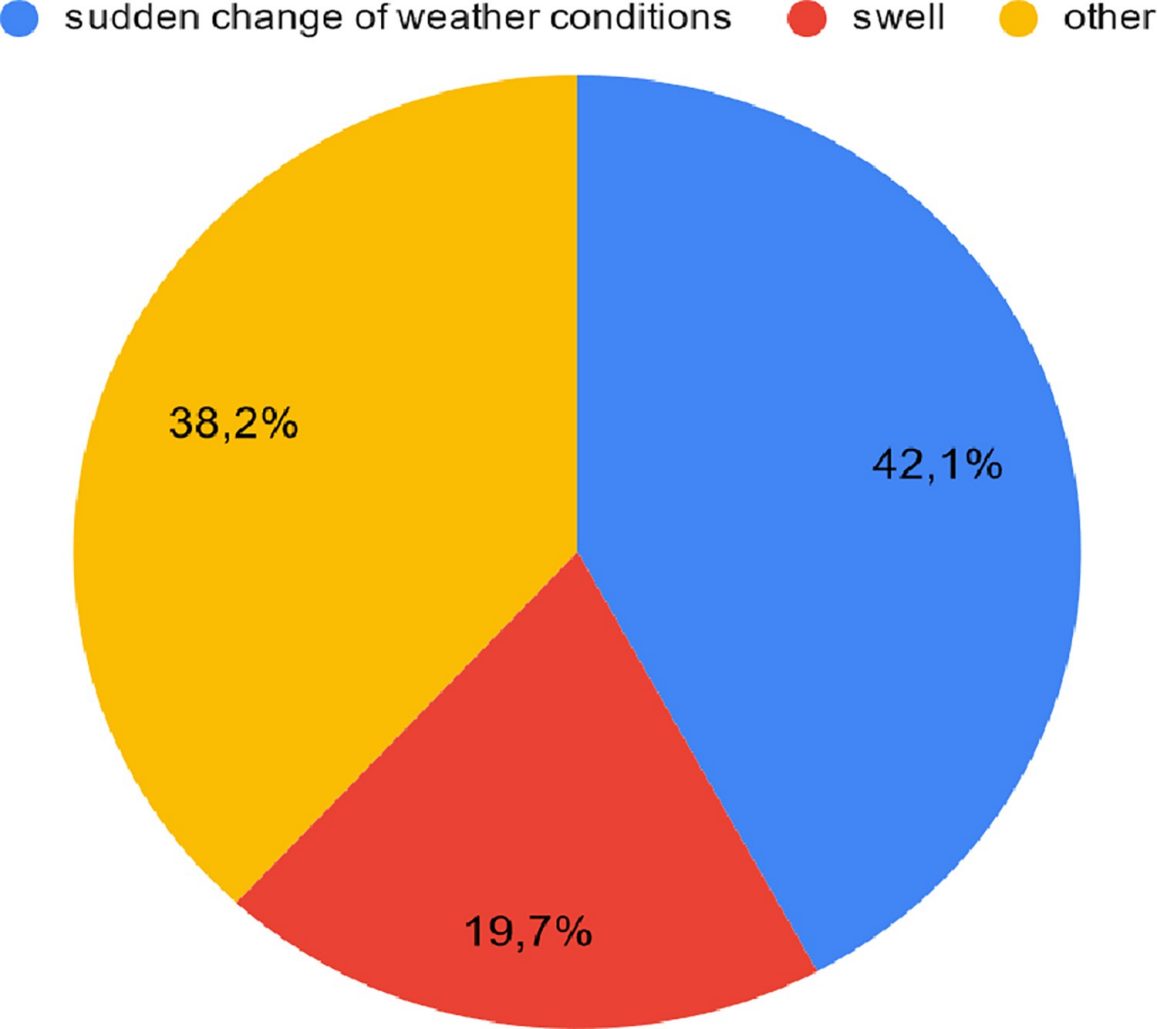
The most common causes of survey equipment failures due to forces of nature according to interviewees.

Sudden change of weather conditions was indicated by 42.1% of respondents as the most common forces of nature contributing to equipment failures. Swell was pointed out by 19.7% of interviewees. Other causes of failures related to the forces of nature were given by 38.2% of respondents (Fig 6).

Examples of other causes of failures related to forces of nature named by the respondents are listed below:

- waving, wind, currents, changing salinity, ice, adverse conditions due to frost, water pressure and temperature;
- refraction;
- misreading of weather forecast;
- taking survey in adverse weather conditions for prolonged periods of time;
- difficult working conditions: water, temperature, vibrations, load;
- failure to properly set up the equipment;
- survey crew not backing themselves to make the correct decision at a time to recover prior to things getting too dangerous for personnel and equipment to be recovered safely in increasing weather;
- poor vessel handling.

#### The most common equipment failures—identified risks and its assessment

After analyzing the documentation and survey responses, the survey equipment failure risks were identified. Later it was assessed by specifying their probability and severity (Table 1). According to the respondents the most frequently mentioned equipment failure is caused by damage to the cable line on which the device is towed. This may be the result of hitting an object lying on the seabed or floating debris and can lead to damage to the probe sensors or even loss of the towed equipment. Interviewees listed many other cable issues, such as mechanical damages (tearing, breakage), twisting the cable in the crane block or in the ship’s propeller. Cable malfunctions cause communication issues with devices. Among the listed reasons there are also leaks, electrical breakdowns at the connectors, damages due to material stress, incorrect maintenance, cuts in the cable insulation causing water ingress and damage to slip rings by salt water and water pressure. Mechanical damage is a frequent group of observed failures. Such may occur among others when equipment hits the seabed or an object lying on it, hits the ship construction or bends on a hard surface when sampling. The respondents also mentioned as common the problems with electricity, electronics and equipment software. It was mentioned that the equipment was damaged when transporting the device or stopping at the port. The respondents also listed a multitude of failures that they remembered the most.

**Table 1:** Classification of identified risks according to Fig 1 (source: Internal data).

No	Risk	Examples	R = P × S	Source of the risk	Actions to reduce risk
1	damage to devices installed on the seabed	Equipment dredged by fishing boat Breaking off the hydro-meteo buoy from the anchor	15 = 3 × 5	human factor forces of nature	Navigation warnings for fishers and local communities Trainings for surveyors
2	loss of towed equipment	Hitting the device against an object at the bottom Hitting the device against the drifting target	15 = 3 × 5	human factor forces of nature	Quality control of the towing cable Including routine equipment check-up to the procedures
3	mechanical damage to the equipment	Hitting the device against an object at the bottom Hitting the device against ship’s side while hauling up/in Hitting the device against the drifting target Bending of the probe due to the hard seabed	12 = 3 × 4	human factor technical factor forces of nature	Trainings for surveyors, Trainings for vessel’s crew Communication between the controller and the operator. Procedures for confirming external conditions / factors for deploying probes into the water. In shallow waters, potentially hazardous locations site surveys should be conducted.
4	collision	Hitting the device against the drifting target Other ship flow on the equipment	10 = 2 × 5	human factor forces of nature	Preparation of survey plan, implementation of safety navigation procedures, using additional vessels at demanding research locations, additional training of operators and vessel crew.
5	hooked or trapped device	Equipment trapped in fishing nets Equipment hooked to a target at the seabed	8 = 2 × 4	human factor	Training of operators, Training of ship crews, mutual communication during equipment set-up. Procedures in the event of equipment being trapped underwater. Navigation warnings for fishers Observation by the helmsman in the event of any fishing nets encounter. Radio communication with fishermen about—measurement activities. Messages in fishing ports
6	blackout during survey / electricity generator malfunction	Vesselwide blackout	8 = 2 × 4	human factor technical factor	Trainings for crew on procedures to restore power in case of blackout
7	cable malfunction	Tearing, Breakage, Cuts in the cable insulation	8 = 4 × 2	human factor technical factor	Storing adequate supply of spare parts on the vessel
8	freezing	Device not adapted to work in low temperatures Water freezes during overflow from the bathometer	6 = 2 × 3	forces of nature technical factor	Staff training, procedures related to labor standards, Training related to health and safety
9	damage to the equipment when transporting the device or stopping at the port	Overturning of poorly secured equipment when heaving	4 = 2 × 2	human factor forces of nature	Developing procedures and standards for transporting equipment from the time of mobilization to the measurement location. Appropriate preparation of transported equipment. Training for equipment operators Paying attention to the sensitivity of individual elements.
10	damage to the equipment against the ship’s propeller	Entanglement / pull-in of the line to which the device is attached to the ship’s propeller	4 = 3 × 3	human factor forces of nature	Development of procedures for starting survey, immersion of equipment. Crew and surveyors trainings Raising awareness of the need for communication, meetings reminding about the conditions, activities and work stages.
11	problems with the software	software crash	3 = 3 × 1	human factor technical factor	Crew and surveyors trainings, Systematic checks of the equipment during mobilization, before reaching the measurement location
12	issues with an oceanographic winch		3 = 3 × 1	technical factor	Adequate equipment servicing procedures, Procedure for checking “dry” equipment during mobilization. Crew and surveyors trainings.

R—Risk, P—Probability, S–Severity.

The failures don’t happen often but have a serious impact on projects due to the enormous waste of time and costs. Among them are collisions when the measurement equipment was destroyed by a support vessel that flowed on the equipment or when the hydrophones broke off due to the impact of a drifting object. Interviewees described incidents when the equipment was trapped into the fishing nets or elements of a shipwreck lying on the seabed. There was a case when it was necessary to call special divers who pulled out equipment trapped at a depth of 60 m. Respondents mentioned problems with devices that are left on the seabed for the purpose of continuous recording of parameters, such as a current profiler. *It happened that the device was flooded*. *The water got in at the very beginning because the gasket was not properly placed*. *A similar measurement attempt ended up even worse*. *The fishing boats dredged our equipment two miles away*. *Most of the equipment was damaged*. *We have only now recovered some parts*.

Respondents also described failures of measuring equipment caused by the forces of nature. The failures listed are most often caused by waves. Most of the damage to the equipment happens when it is brought onto the deck of the vessel. When the vessel is rocked, it is difficult to control heavy equipment hanging on a cable. The devices suffer mechanical damage from impacts on the ship’s structure. In worst-case scenarios big waves stress the cable, which often breaks off and results in equipment sunk (*Once we lost the entire 6 km hydrophone cable*. *It was a very windy night*, *the sea was rough and maybe the cable was not properly tied*). The device loss may also occur due to poor securing of equipment on board the vessel, which may fall overboard by swinging *(A blow of wind pushed the vibrocore onto the safety chains on the stern*, *2 cm-thick chain broke*). The respondents also indicated problems with the equipment due to low temperatures, such as freezing of equipment, freezing of water in a bathometer. Also the impact of phenomena on the quality of measurement data like refraction and magnetic storms was mentioned. Hydroacoustic data acquisition during unfavourable weather conditions also affects their quality.

## Discussion

### Reference to the main research objectives

The aim of the research was to identify the most common measuring equipment failures while working at sea, find its causes according to results of conducted surveys, assess and quantify risks. We also proposed a response plan for each identified risk.

### Summary of the main findings of the article

Twelve basic equipment failure risks were identified in our paper based on the review of relevant project documentation (DPRs, Observation Cards) and information-gathering techniques (questionnaire). After analyzing the documentation, the risks were divided into three main sources: human factor, technical factor and forces of nature. The questionnaire was designed to obtain as much information as possible about the types of failures with examples and their causes according to these three groups. We Authors assessed the risk in terms of the likelihood of its occurrence and its consequences on the project schedule creating a risk matrix. Each risk was calculated as a combination of potential hazard severity and probability of occurrence of this hazard and assigned to the appropriate zone: green (acceptable risk), yellow (significant risk) or red (unacceptable risk).

The group of the unacceptable risk includes: damage to devices installed at the seabed, loss of towed equipment, mechanical damage to the equipment and collision. Among the sources for the listed risks are all three: human factor, technical factor and forces of nature. Two failures of the biggest calculated risk have probability at the level up to 40% with a very high impact on the work schedule which could be even more than 2 weeks delay although, based on the responses of our interviewees, delays can be counted in months. Many studies [35, 43] require devices like Acoustic Doppler Current Profilers (ADCP) or Acoustic Wave and Current Profiler (AWAC) to be installed at the seabed in order to continuously record parameters such as currents or hydrophysical parameters. Problems that may arise during the survey include data recording errors, damage or even complete loss of the device.

Interviewees mentioned that one of the causes of the total hardware loss was dredged equipment installed at the seabed by the fishing boats. However, placement of any research buoy such as a profiling buoy or weather buoy in the Polish exclusive economic zone should be reported to the Hydrographic Office of the Polish Navy that publishes each week ‘*Notices to Mariners*’. The publication lists new potential obstacles or obstructions to safe navigation which are then included in the electronic and paper maps which are available to sea users. Yet, not every map service or publication is updated regularly or the updates are not checked by the users. Therefore, as mentioned before, loss of research equipment may happen.

**Fig 7:**
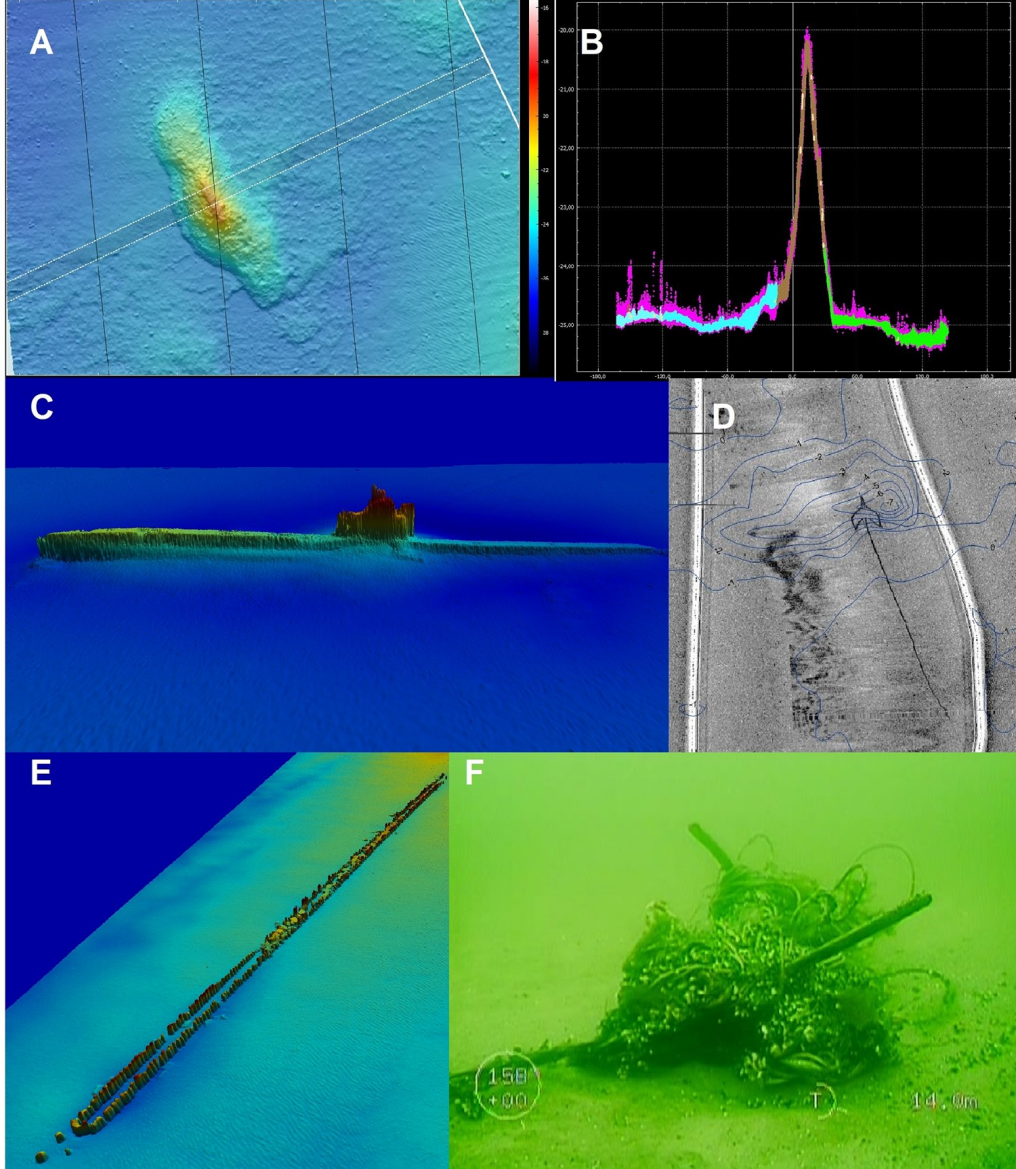
Examples of objects on the seabed which may interfere with the survey: A) geological form on the seabed, image from the multibeam echosounder (MBES), B) single MBES profile in the location of the geological form A—height 5 m above the seabed, C) wreck of the Ślązak vessel, MBES image, D) fishing nets at the seabed of the reservoir, side-scan sonar SSS image with magnetic field anomaly lines, E) Palisade remains, MBES image, F) abandoned fishing nets, ROV TV picture.

Loss of towed equipment was also one of the most frequently mentioned failures. Such an event is another risk associated with a large loss of time for the project and increased costs. The search for a device in some cases can take several days and it is not always successful. The most common cause of loss of towed equipment is a collision with an obstacle at the bottom in the survey area (Fig 7).

**Fig 8:**
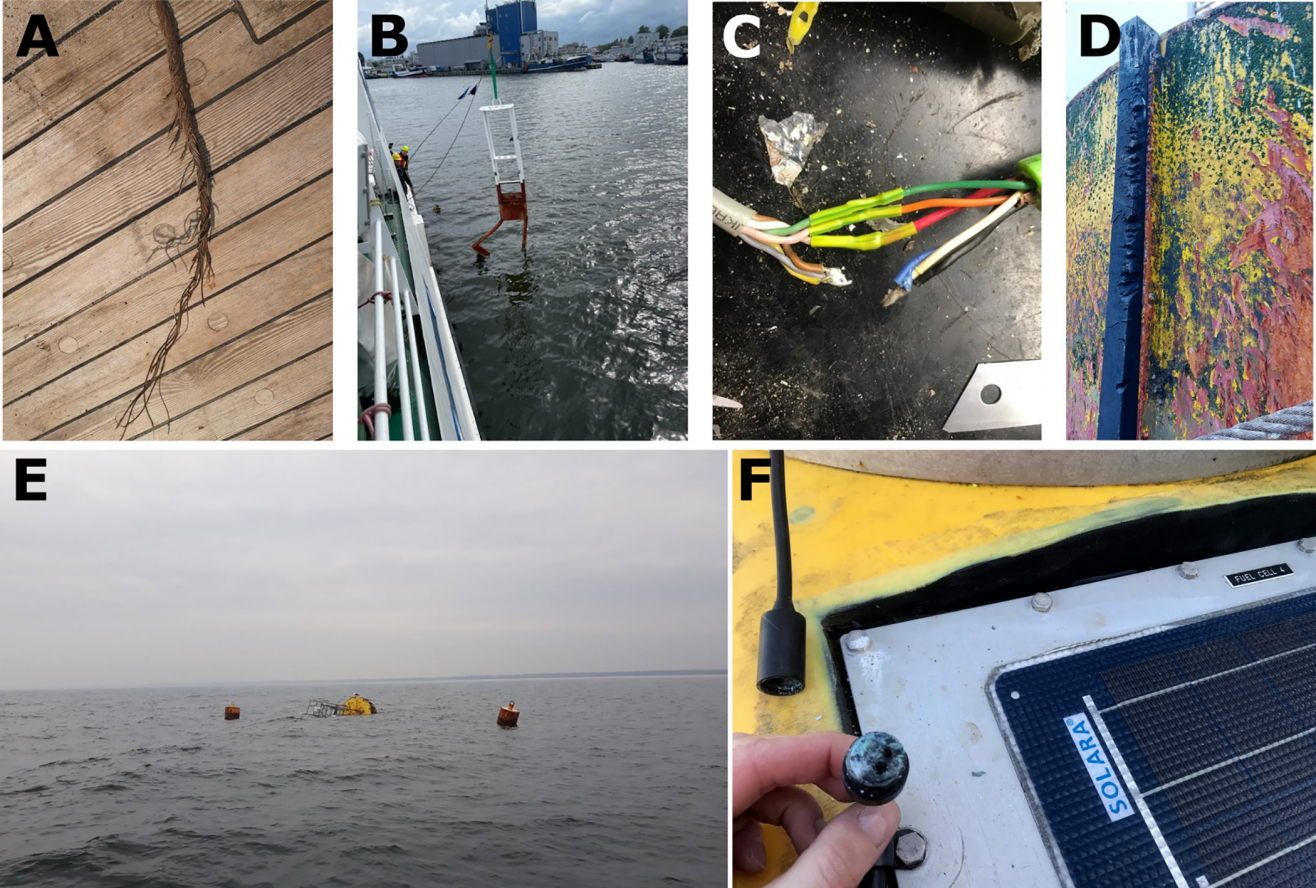
Examples of mechanical damage. A) Steel rope of the ship’s crane broken when lifting a device from the seabed, B) Hitting the MBES frame on the underwater installation, which resulted in a deformation of the frame and loss of the device, C) Broken cable, D) Damage to the measuring buoy plating due to a collision with another floating object, E) Tipping over of the measurement buoy after breaking the anchor due to severe weather conditions, collision / trampling by other floating object / lack of appropriate services, F) Damaged cable.

When choosing the survey method, impact of environmental conditions on imaging accuracy by using hydro-acoustic systems [44] should be taken into account especially in waters of a high non-uniformity of spatial distribution of hydrological parameters. Complex environmental conditions in shallow sea, especially changeable seasonal temperature distribution which directly affects spatial distribution of sound propagating in water column [45], are of a great importance for accuracy in seabed imaging. To avoid the impact of refraction on data quality the device is towed close to the seabed, which may result in hitting the seabed or an obstacle. Here the operator’s caution and experience are of great importance. Another type of survey where the device is towed over the bottom is a magnetometer survey. The maximum size of an iron object which can be detected is determined by the distance from the object to the magnetometer [46]. The two main survey parameters which affect this distance are the altitude of the magnetometer above the seabed (and target) and the distance between survey run lines [47, 48]. In order to detect smaller targets it is desirable to tow the magnetometer towfish as close to the seabed as possible. As a consequence, there is a danger of hitting the seabed or the target with the towfish and appropriate distance between the seabed and the device must be maintained which will depend on the nature of the seabed itself, the prevailing sea conditions and the courage of the operator [47]. Hitting the seabed or target with the towfish may cause a breakage of the cable and equipment loss, but also may affect mechanical damage. Based on our analyzes, other causes of such damage are also hitting the device against ship’s side while hauling up/in, hitting the device against the drifting target or bending of the probe due to the hard seabed (Fig 8).

**Fig 9:**
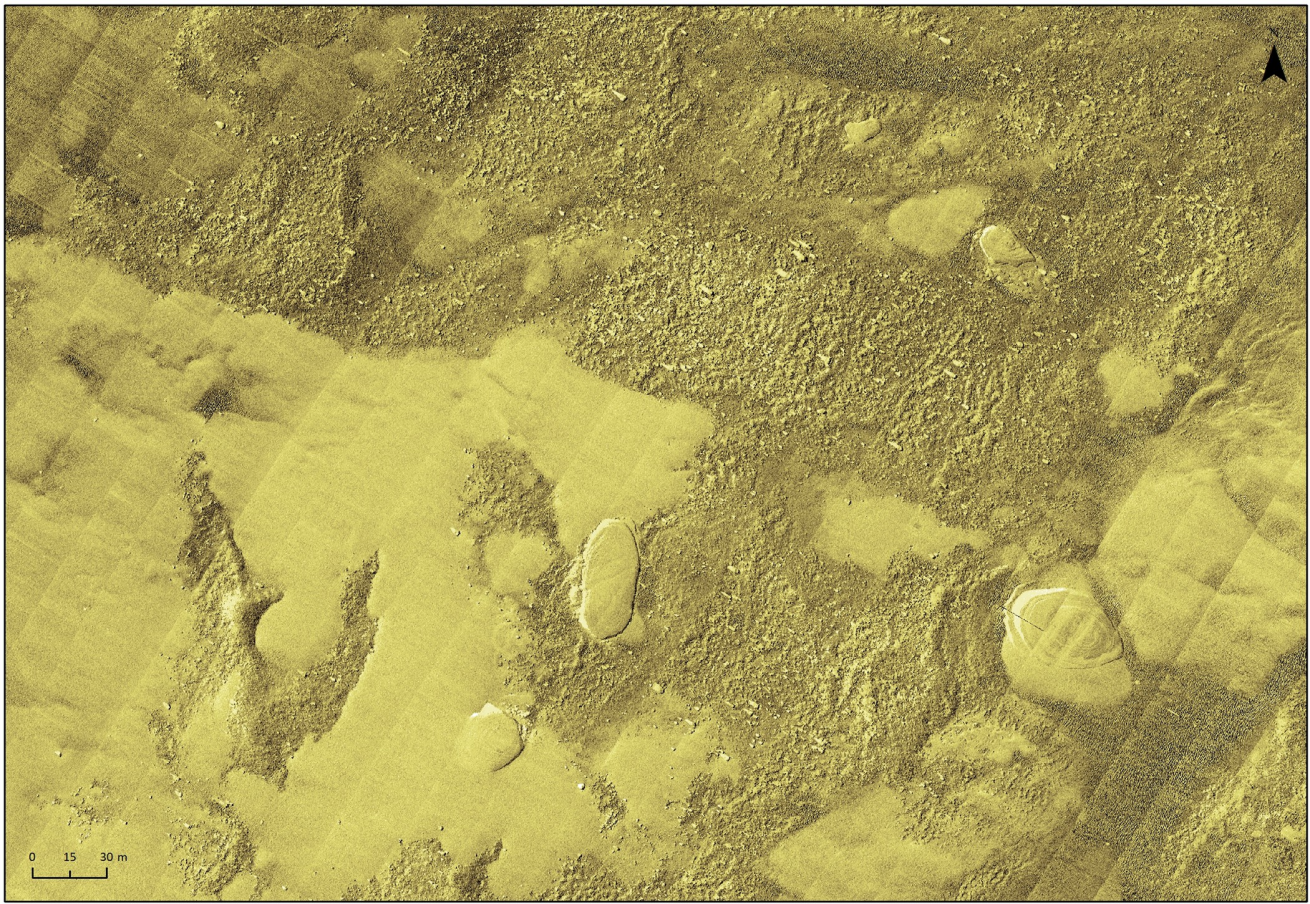
Side-scan sonar image of seabed consisting of various sediment types.

The last example may occur when sampling or coring is carried out on the bottom covered with various types of sediments (Fig 9) as it can not be operated in rocky substrates [49]. In this case, the best way to avoid a failure is to pre-identify the bottom surface and then plan the sampling. Risks of mechanical damage to the equipment according to the risk matrix was estimated at 12 with highly likely probability more than 40% and high consequences for the project.

Collisions like hitting the device against the drifting target or other ship flow on the equipment were assessed as unlikely but with very high consequences to the project schedule. The probability of an event occurring may be low, but if it does, the consequences can be catastrophic and result in complete loss/damage of equipment.

The group of the significant risk includes: hooked or trapped device, blackout during survey, cable malfunction, freezing. All of these risks have been assessed as very unlikely except for cable malfunction which has a highly likely probability of occurrence and its risk severity was assessed as low as repair usually doesn’t take long but only if there are spare parts onboard and people with appropriate knowledge and skills. In the survey, we found descriptions of the accidents when the device was trapped into the fishing nets or elements of a shipwreck. The events were mentioned as those that were most memorable for the respondents, so we conclude that they caused a lot of trouble for project participants. Such accidents happen when the area under investigation is not well recognized or there are surprises such as recently deployed fishing nets without navigation warnings. The key factor here is the caution of the equipment operator. Problem of freezing depends primarily on the research region as well as the season. In some campaigns the risk will not be taken into account at all, while in some projects this phenomenon can significantly delay work.

The group of the acceptable risk includes: problems with the software, damage to the equipment when transporting the device or stopping at the port, damage to the equipment against the ship’s propeller, issues with an oceanographic winch. These occurrences are assessed as probable or unlikely with moderate, low or very low consequences to the project. However, these problems cannot be ignored. The human factor appears as the source of risk in almost all cases. The basis for success is therefore good work organization and a backup plan to each potential risk. Moreover analyzing the above, it becomes clear that the risks identified as unlikely occurring individually have little consequences for the project implementation. The effects can be serious, when the accumulation of risks occurs or when we sum up their occurrence.

The results of our survey conducted among people involved in work on research vessels indicate that the most common cause of failure of measuring equipment is human factor. It is generally stated that 80% of all accidents at sea are a result of human error [50, 51] however, Wróbel [52] gave this statement a broad analysis and claims it unsubstantiated. Moreover the literature mainly refers to accidents at sea, but not every failure of a measuring device can be classified as an accident and the cases examined in the literature do not only concern equipment installed on board. To our best knowledge, this work is the first one that undertook the identification of measuring equipment failures risks in offshore surveys. According to our respondents the most common cause of survey equipment failures was assessed as a human factor, the second important cause was a technical factor, with the main cause of the failure indicated as poor technical conditions of tools or instruments. According to DNV [53] failure analysts most commonly use four general descriptions of failure damage mechanisms: fracture, corrosion, wear, and distortion (or undesired deformation). Finding physical root cause is vital action to avoid a problem reoccurring. Such analysis also requires an interdisciplinary approach, according to Edwards [54] three levels: physical roots, human roots and latent roots (procedural, organizational in nature, environmental or other beyond the realm of control). Among the risks associated with the forces of nature, in the case of offshore works, weather is the greatest [11]. According to our survey, sudden change of weather conditions was indicated by 42.1% of our respondents as the most common force of nature contributing to equipment failures. Good weather risk management can be a tool to avoid a range of potential failures. Although when analyzing the collected research material, an important conclusion is clearly visible: the relationship between the occurrence of equipment failures and the possibility of avoiding them by improving the quality of project management. Many of the event descriptions explicitly or implicitly indicated that failures could have been avoided by more appropriate planning of the survey process (choice of equipment, choice of personnel, choice of research method). Such relationships can be found in each of the three groups of factors causing equipment failures.

### Limitations of our research

Due to the nature of the offshore marine research vessel industry, it is extremely difficult to gain access to suitably qualified and experienced surveyors and project managers willing and able to participate in this type of research. The small size of this particular population sample does not allow generalizations and as such, the results of this survey should not be seen as representative of the trends dominating in the entire industry.

As safety-related matters are a very important factor in this industry, and are often considered sensitive, the offshore marine companies are reluctant to allow researchers access to the safety-related data which they possess, due to confidential clauses in their contracts.

### Recommendations for future research

Presented results of the analysis on equipment failures do not exhaust the subject matter at hand. On the contrary, several questions arise that may prompt additional scientific research undertakings. The sources of equipment failures were divided into human and technical aspects as well as those resulting from the unpredictability of nature. Therefore, it would be worth exploring how the staff of survey vessels for a particular research operation is recruited, what are the qualifications and conditions they need to meet, how the research is organised and managed, how the human risk is accounted into planning and implementation of a project during its lifetime. And on top of that, how cost efficiency or cost cutting determines selection of staff, equipment, organisational structure and procedures when arranging a research endeavour at sea and if it significantly influences risk of equipment failures. How unpredictability of the weather conditions is factored in the research schedule, are weather risks avoidance procedures in place? What is the financial cost of weather-related delays on the marine research that is a part of an offshore investment project? Could there be proposed any new legislation facilitating introduction of risk management procedures minimising risk of equipment failure and therefore, reducing delays in the realisation of much needed investments in offshore energy?

To answer these questions, however, a wider examination needs to be conducted, with the application of methods that go beyond the information contained in the reports. Further analysis on a much larger scale based on wider scope of information from documents and experts should provide enough material for more knowledge and experience on effective management of risks arising from equipment failure.

## Conclusions

Investigation of the marine environment is a complex process that requires involvement of appropriate resources of equipment and people. The main purpose of this paper was the identification of factors that may adversely affect work on board research vessels. Among the three main elements which we identified during the documentation review were human factor, technical factor and forces of nature. A survey addressed to people involved in the implementation of offshore projects indicated that the most common cause of survey equipment failures was human factor. The analysis of the remaining factors also showed that in some way they are all related to human factor.

The fact that 76.3% respondents participated in a project which had to be stopped due to a failure of the survey equipment which required return to the port shows that the problem which we analyze is of particular importance for efficient work at sea. Crucial element that restricts implementation of offshore projects is unfavourable weather conditions which are beyond human control. Thus, the time in which we can conduct research is limited and the accessibility of the research areas should be used as best as possible. Therefore, proper risk management is necessary.

The list of twelve identified survey equipment failure risks in the paper is far from being exhaustive but it seems universal. Increasing awareness among management and employees will reduce the number of unforeseen events and the severity of their consequences. As a result, it also allows effective protection of the resources, as well as reducing risk costs and work schedule extension. The authors also provide examples of actions to reduce identified risks. Such a backup plan is a mandatory part of a good risk management plan.

Our analyses have revealed a long list of potential failures that may occur during the research work onboard the ship, which has not been presented before. Collecting information on failures that have occurred so far in implemented projects helps to determine approaches that can be taken to investigate why the failure has occurred and how to prevent it in the future. The article also highlights the relationship between the quality of research project management and its implementation in the context of the failure rate of measuring equipment. We came to the conclusion that the most important element at every stage of the project implementation are people and decisions made.

## Supporting information

S1 Data (XLS)

S1 File Questionnaire: Failures of measuring equipment. (PDF)
