# Supplementary material for: Many faces of survey equipment failures during marine research at sea—Risk analysis
Source: PLoS One. 2022 Aug 26;17(8):e0272960. doi: 10.1371/journal.pone.0272960 (PMC9422978; doi:10.1371/journal.pone.0272960)
Supplement: S1 File — (PDF) [file pone.0272960.s002.pdf]

Questionnaire: failures of measuring equipment

1. You are employed as?

- Surveyor
- Crew member
- other: ______________

2. How long have you been working at sea?

- less than 1 year
- 1-5 years
- 5-10 years
- over 10 years

3. Have you participated in a project works in which had to be stopped due to a failure of the research vessel?

- Yes
- No

4. Have you participated in a project the works in which had to be stopped due to a failure of the measuring equipment requiring a return to the port?

- Yes
- No

5. Describe the most common measuring equipment failures you have encountered while working at sea:

____
____
____
____
____
____

6. Describe the most memorable equipment failure onboard a vessel that you have witnessed. What caused the failure?

____
____
____
____
____
____

7. What do you think is the most common cause of measuring equipment failures? (assess factors from 1 - the least common to 5 - the most common)

	1	2	3	4	5
human factor	◯	◯	◯	◯	◯
forces of nature	◯	◯	◯	◯	◯
technical factor	◯	◯	◯	◯	◯

8. What do you think is the most common human factor contributing to equipment failures (you can choose multiple answers)
    - lack of caution
    - lack of qualifications or good training
    - rush
    - no compliance with the procedures
    - fatigue
    - poor work organization
    - other________________________

9. What do you think is the most common technical factor contributing to equipment failures?
    - inadequate tools or instruments
    - tools or instruments in poor technical condition
    - other________________________

10. What do you think are the most common forces of nature contributing to equipment failures?
    - sudden change of weather conditions
    - swell
    - other________________________

11. List/describe the failures of measuring equipment caused by the forces of nature that you have witnessed:

____

____

12. In which sea basins were the projects you have participated in implemented?

____

____

____

Thank you for your time! Please share this questionnaire with your colleagues.
